# Multisystem Inflammatory Syndrome Causing Mixed Cardiogenic Shock in a 34-Year-Old With Influenza A

**DOI:** 10.7759/cureus.36417

**Published:** 2023-03-20

**Authors:** Simon Kashfi, Matthew Pierce

**Affiliations:** 1 Internal Medicine, Zucker School of Medicine at Hofstra/Northwell Health, Manhasset, USA; 2 Cardiology, Zucker School of Medicine at Hofstra/Northwell Health, Manhasset, USA

**Keywords:** ivig, thermodilution, influenza a myocarditis, cardiogenic shock, mis-α

## Abstract

Multisystem inflammatory syndrome in adults (MIS-A) is a sequela of COVID-19 and can cause mixed cardiogenic and vasodilatory shock. We present the case of a 34-year-old female who presented with mixed cardiogenic and vasodilatory shock and was found to be influenza A positive while also meeting criteria for MIS-A. She responded well to treatment with steroids and intravenous immunoglobulin (IVIG).

## Introduction

Viral infections are the most common causes of myocarditis and are responsible for 50-70% of all myocarditis cases [1]. Of the influenza viruses, influenza A is associated with the highest cardiovascular morbidity and mortality [1]. Nearly 10% of cases of influenza, in general, can present with a clinical diagnosis of acute myocarditis, and rates increase to nearly 40% on autopsy [2]. The clinical presentation of myocarditis is variable, ranging from no symptoms to cardiogenic shock [3]. However, presentation with cardiogenic shock is rare and is known as fulminant myocarditis [1]. The diagnosis of viral myocarditis is clinical but can also be supported with an endomyocardial biopsy. Multisystem inflammatory syndrome in adults (MIS-A) is a rare and increasingly recognized sequela of SARS-CoV-2 infection. It was first described by the Centers for Disease Control and Prevention (CDC) in October 2020 through a review of three case series and several case reports [4]. Multisystem inflammatory syndrome had already been recognized in children, and the adult form was described later [5]. Of the three case series reviewed, the one by Chau et al. was the first to describe patients presenting with cardiogenic shock [6]. We present the case of a 34-year-old female who presented with cardiogenic shock and was found to be influenza A positive while also meeting criteria for MIS-A.

This article was previously presented as a meeting abstract/poster at the ACC.23 Annual Scientific Session & Expo on March 4, 2023 [7].

## Case presentation

**Figure 1:**
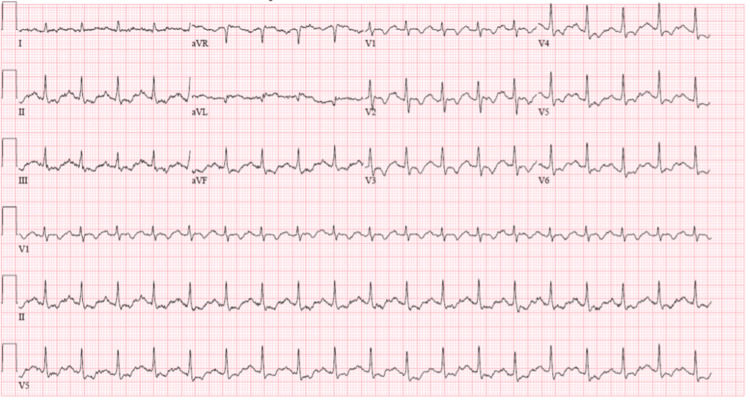
Electrocardiogram on admission.

The patient is a 34-year-old female with a history of adrenal insufficiency (unknown during hospital course and not taking any medications) and hypothyroidism on levothyroxine who presented to an outside hospital after being found down and unresponsive at home. She had a cough and weakness the preceding days. Her initial vital signs were notable for a temperature of 103F, blood pressure of 82/64 mmHg, heart rate of 115 beats per minute, and hypoglycemia. On the physical exam, the patient smelled of urine. Cardiovascular exam was notable for tachycardia with no murmurs. Lungs were clear to auscultation bilaterally. Extremities were warm. She was started on a norepinephrine infusion (after receiving 3L of intravenous fluids). Point-of-care ultrasound showed severe biventricular dysfunction. The left ventricular outflow tract velocity time integral was 8.1 cm. Notable initial labs are shown in Table 1. She was also found to be influenza A positive on polymerase chain reaction testing. Electrocardiogram on admission showed sinus tachycardia with ST and T wave abnormalities (Figure 1). A computed tomography scan angiogram of the chest was negative for pulmonary embolism. She was then transferred to our institution for further management.

**Table 1:** Notable lab values on admission.

Test (reference range, units)	
Creatinine (0.50-1.30, mg/dL)	1.89
Troponin I (<53.7, ng/L)	2453
Lactate (0.5-2.0, mmol/L)	5.1
D-dimer (<229, ng/mL DDU)	806
C-reactive protein (0-4, mg/L)	142
Procalcitonin (0.02-0.10, ng/mL)	26.83
Ferritin (15-150, ng/mL)	1207
Brain natriuretic peptide (BNP) (0-300, pg/mL)	14,487

Right heart catheterization on 2 ug/kg/min of norepinephrine showed a right atrial pressure of 11 mmHg, right ventricular pressure of 34/13, pulmonary artery pressure of 35/25/24 mmHg, and a pulmonary capillary wedge pressure of 11 mmHg. Cardiac index (CI) by Fick was 2.82 L/min/m^2^ and the systemic vascular resistance (SVR) was 300 dynes/sec/cm^−^^5^. These values were more consistent with distributive shock, and mechanical support was not placed. An endomyocardial biopsy was deferred at this time. An echocardiogram performed before cardiac catheterization showed grossly low-normal left ventricular systolic function (ejection fraction 52%), right ventricular enlargement, and decreased right ventricular systolic function. The patient was then transferred to the cardiac intensive care unit (CICU). A second echocardiogram was performed, which showed grossly moderate global left ventricular systolic dysfunction (ejection fraction 40%), right ventricular enlargement with decreased right ventricular systolic function.

After transfer to the CICU, she was started on oseltamivir as recommended by an infectious disease specialist. Despite the high dose of norepinephrine and normal CI, her hemodynamics worsened, requiring the addition of vasopressin. She began to have respiratory distress requiring bilevel positive airway pressure (BIPAP). Her lactic acidosis also worsened and was resuscitated for her acidosis with aggressive bicarbonate repletion. Her peak lactate was 8.1 mmol/L, and pH on arterial blood gas prior to initiating bicarbonate supplementation was 7.27. Due to her high pressor requirement, a stress dose of steroids with hydrocortisone was initiated. CI by the Fick method (now using a pulmonary artery catheter) remained within normal limits, but lactate continued to rise. Thermodilution was then performed, which showed a CI of 1.6 L/min/m^2^ and an SVR of 2200 dynes/sec/cm^−^​​​​​​​^​​5^, indicating more of a cardiogenic etiology of shock. The patient was then started on dobutamine, and her doses of norepinephrine and vasopressin were slowly weaned.

The following day, the patient developed severe abdominal pain and was found to have elevated COVID-19 spike domain and nucleocapsid antibodies, meeting criteria for MIS-A. The infectious disease specialist recommended to start a five-day course of intravenous immunoglobulin (IVIG). Over the next few days, while completing her course of IVIG, the patient was weaned off of vasopressors and inotropes, though she continued to have discordance between her Fick and thermodilution CIs. An agitated saline study was negative for a patent foramen ovale. Vasoactive medications were stopped on hospital day four. On hospital day five, she had a cardiac MRI, which showed no focus of late gadolinium enhancement to suggest fibrosis or scarring. A calculated ejection fraction was 50.84% with an enlarged right ventricle. She was then transferred to the floor with isosorbide dinitrate and hydralazine for afterload reduction. After a few days on the floor, isosorbide dinitrate and hydralazine were transitioned to spironolactone, dapagliflozin, and sacubitril/valsartan. A beta-blocker was trialed but not tolerated due to bradycardia. She was discharged home on a steroid taper. At a follow-up cardiology visit three weeks later, she reported being able to walk one block before becoming short of breath and was euvolemic on the exam. One month later, the patient had a repeat echocardiogram, which demonstrated a recovered left ventricular ejection fraction of 60% and a normal right ventricle. BNP had also returned to normal.

## Discussion

**Figure 2:**
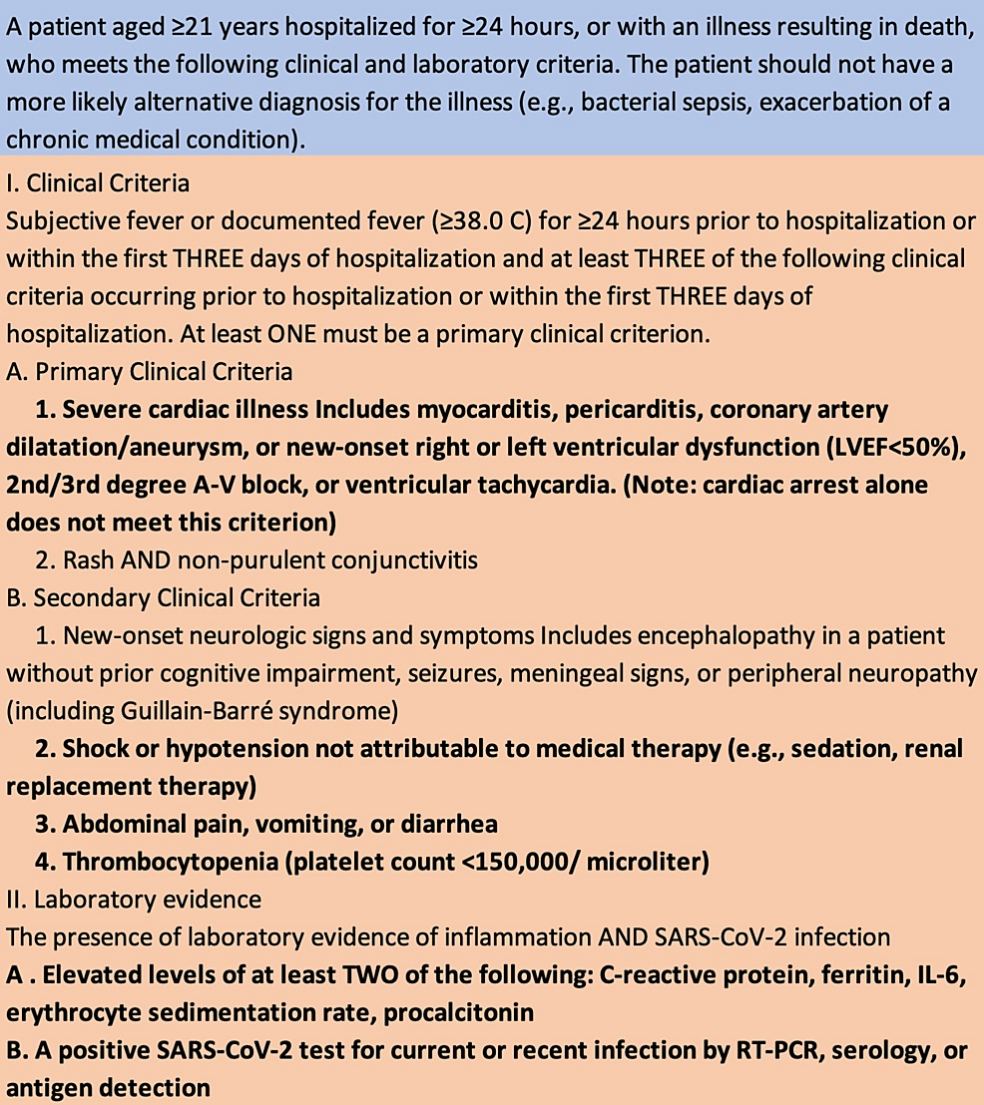
CDC case definition for MIS-A. Criteria fulfilled by the patient are in bold. IL-6: interleukin-6; RT-PCR: reverse transcriptase-polymerase chain reaction; CDC: Centers for Disease Control and Prevention; MIS-A: multisystem inflammatory syndrome in adults.

This case involves a young patient with no prior cardiac history presenting with mixed cardiogenic and vasodilatory shock. Unique to our case was that the patient had a combination of influenza A and MIS-A. The question arose as to which entity was driving the patient’s presentation. Of note, influenza myocarditis is a clinical diagnosis, with criteria including at least two of the following: fever, chest pain, paradoxical pulse, or increased heart size, plus at least one of the following: EKG findings consistent with myocarditis, a 4× increase in IgG antibody titers, or pericardial effusion [1]. This patient did not meet these criteria. The European Society of Cardiology also offers diagnostic criteria for clinically suspect myocarditis, which this patient did not meet, specifically because of the lack of imaging findings on cardiac MRI [8]. Additionally, the Lake Louise criteria can be used to diagnose myocardial inflammation on cardiac MRI [8,9]. This criteria, updated in 2018, suggests that myocardial inflammation is present if there is myocardial edema on T2 imaging or either late gadolinium enhancement, T1 mapping, or increased extracellular volume on T1 imaging. Though having both T1 and T2 findings increases the specificity, both are not required for diagnosis. Regardless, this patient’s cardiac MRI did not reveal any of these findings. Rather, our patient’s rapid improvement after starting IVIG and steroids was more suggestive of MIS-A. Our patient met the diagnostic criteria for MIS-A, as described by the Centers for Disease Control and Prevention (CDC) (Figure 2) [10].

Patients with MIS-A present a median of 28 days after SARS-CoV-2 infection with fever, hypotension, cardiac dysfunction, gastrointestinal, and respiratory symptoms. The vast majority have elevated inflammatory markers [11]. Additionally, patients are mostly young with little to no comorbidities. This patient’s initial right heart catheterization showed a mixed cardiogenic and distributive shock. While on 2 ug/kg/min of norepinephrine, the patient had an SVR of 300 dynes/sec/cm^−5^, indicating significant vasodilation. Additionally, she had elevated right-sided pressures, indicating right ventricular failure. This pattern of mixed shock, along with rapid improvement when treated for MIS-A, was seen in other reported cases of MIS-A [6,12-17]. The pathophysiology of heart failure in MIS-A is uncertain and may be due to myocardial stunning in the setting of a dysregulated immune response [11,15]. The propensity of MIS-A for young and middle-aged adults might be explained by their stronger immune systems when compared to older adults and the elderly [11].

The decision to start dobutamine was based on the thermodilution measurement of the cardiac index. This measurement fit the patient’s clinical picture more so than the CI by Fick. Her initial CI, normal by Fick, was likely an over-estimation and would have been much lower and consistent with cardiogenic shock were it calculated by thermodilution. While the two calculations should be within range ideally, it is not uncommon for them to be discordant. Thermodilution can be lower than Fick in the setting of right ventricular dysfunction. Opotowsky et al. compared the two methods in a retrospective study with more than 15,000 patients [18]. The authors found that the estimates of cardiac output differed by >20% in 38.1% of all catheterizations. Additionally, they found that thermodilution better predicted all-cause mortality and was, therefore, considered more accurate than the Fick method. The study did not stratify by the presence of right heart failure. 

There is no gold standard of treatment for MIS-A, and treatment for the adult form has been adapted from its pediatric counterpart. The general approach is to initiate corticosteroids, intravenous immunoglobulin, and in some cases, cytokine inhibitors [17,19]. Our patient saw significant improvement after the initiation of hydrocortisone and IVIG and was weaned off of all support with improved biventricular function.

The case is limited by the lack of an endomyocardial biopsy for the evaluation of influenza myocarditis. Though the patient did not have the clinical or imaging criteria for influenza myocarditis, a biopsy is still the gold standard. However, even if a biopsy had been performed, the biopsied site may not have shown active disease [1].

## Conclusions

MIS-A is a rare sequela of the SARS-CoV-2 infection and can cause a mixed cardiogenic and distributive shock. Providers should take a detailed infection history and check COVID serologies in those presenting with mixed shock, especially younger patients and those without significant cardiac history or risk factors. The diagnosis and treatment of MIS-A should be coordinated with infectious disease consultants. Corticosteroids and IVIG are the most acknowledged treatment options to which patients have responded well.
